# Anti‐oxidative and anti‐inflammatory effects of Ginkgo biloba extract (EGb761) on hindlimb skeletal muscle ischemia–reperfusion injury in rats

**DOI:** 10.14814/phy2.16050

**Published:** 2024-06-05

**Authors:** Liang‐Yi Chen, Shih‐Huang Tai, Yu‐Chang Hung, Sheng‐Yang Huang, Zi‐Cheng Kuo, Ai‐Hua Lee, Hao‐Hsiang Hsu, Tian‐Shung Wu, E‐Jian Lee

**Affiliations:** ^1^ Neurophysiology Laboratory, Neurosurgical Service, Department of Surgery, National Cheng Kung University Hospital, College of Medicine National Cheng Kung University Tainan Taiwan; ^2^ School of Pharmacy, College of Medicine National Cheng Kung University Tainan Taiwan

**Keywords:** anti‐inflammatory, anti‐oxidative, Ginkgo biloba, ischemia, skeletal muscle

## Abstract

In posterior spine surgery, retractors exert pressure on paraspinal muscles, elevating intramuscular pressure and compromising blood flow, potentially causing muscle injury during ischemia–reperfusion. Ginkgo biloba extract (EGb 761), known for its antioxidant and free radical scavenging properties and its role in treating cerebrovascular diseases, is investigated for its protective effects against muscle ischemia–reperfusion injury in vitro and in vivo. Animals were randomly divided into the control group, receiving normal saline, and experimental groups, receiving varying doses of EGb761 (25/50/100/200 mg/kg). A 2‐h hind limb tourniquet‐induced ischemia was followed by reperfusion. Blood samples collected pre‐ischemia and 24 h post‐reperfusion, along with muscle tissue samples after 24 h, demonstrated that EGb761 at 1000 μg/mL effectively inhibited IL‐6 and TNF‐α secretion in RAW 264.7 cells without cytotoxicity. EGb761 significantly reduced nitric oxide (NO) and malondialdehyde (MDA) levels, myeloperoxidase (MPO) activity, and increased glutathione (GSH) levels compared to the control after 24 h. Muscle tissue sections revealed more severe damage in the control group, indicating EGb761's potential in mitigating inflammatory responses and oxidative stress during ischemia–reperfusion injury, effectively protecting against muscle damage.

## 1 INTRODUCTION

Posterior spinal surgery often results in weakness of the paraspinal muscles after the surgery which was regarded as reason of postoperative pain, adjacent segment disease, and kyphosis (He et al., 2020). In addition to the damage caused by muscle detachment, the temporary ischemia–reperfusion can be caused by the retraction device used during the surgery (Cho et al., 2020).

Previous studies have indicated that the use of drugs with free radical scavenger properties, such as edaravone, can reduce ischemia–reperfusion injury in skeletal muscle in animal models (Hori et al., 2013). Ischemia–reperfusion injury produces excessive oxygen free radicals and inflammatory mediators, caused neutrophils accumulated in the local and circulation. These mediators cause endothelial cell dysfunction and cellular damage. Therefore, that reduce of free radicals, could reduce the tissue damage caused by ischemia–reperfusion.

Ginkgo is a natural product purified from Ginkgo bilobae tree. Previous studies have shown that EGb761 has antioxidant, anti‐inflammatory, and free radical scavenging abilities (Barbalho et al., 2022). Therefore, this study hypothesizes that administering EGb761 prior to inducing ischemia–reperfusion in an animal model could simulate and mitigate the damage caused to skeletal muscles, potentially offering a protective mechanism against such injuries. By utilizing its abilities to scavenge free radicals, reduce inflammation, and provide antioxidant effects, the aim is to determine whether EGb761 can reduce the degree of muscle injury.

## 2 MATERIALS AND METHODS

### 2.1 Ginkgo biloba extraction

For extraction, 1 g of dry Ginkgo leaves was extracted three times with 30 mL of 70% MeOH by sonication for 30 min. The extracts were combined and evaporated to dryness using a rotary evaporator. The crude extract was dissolved in 30 mL of 5% HCl, heated to reflux for 30 min, and then partitioned three times with cyclohexane (30 mL). The remaining water layer was partitioned by methylethyl ketone (30 mL × 3 times). The methylethyl ketone fractions were evaporated and dissolved in 0.6 mL of a mixture of methanol‐d4 and benzene‐d6 (65:35). Ginkgo product were dissolved in 30 mL of 5% HCl for hydrolysis and processed as described above. The recovery test of reference compounds was according to Choi's method (Choi et al., 2003). One gram of filter paper disks (5892 white ribbon ashless, Schleicher & Schuell, GmbH, Cassel, Germany) was cut into a 1 cm diameter and placed in the extraction vessel. Each standard of bilobalide; ginkgolides A, B, C, and J; kaempferol; quercetin; and rutin (3.0 mg) was spiked into the filter paper disks. Then, the spiked samples were dried in a vacuum oven at 40°C for 24 h. These cellulose papers were extracted, hydrolyzed, processed as described above, and quantified by the 1H NMR method.

### 2.2 Chemicals and instrument

Cyclohexane and methylethyl ketone were purchased from Merck (Darmstadt, Germany). Methanol‐d4 (99.8%), benzene‐d6 (99.6%), and 1,3,5‐trimethoxybenzene were obtained from Sigma‐Aldrich (St Louis, MO, USA). 1H NMR spectra were recorded using the Bruker AV Ascend^TM^ 400 MHz (Bruker, Billerica, MA, USA) NMR spectrometer. For each sample, 100 scans were recorded with the following parameters: 0.187 Hz/point; spectra width, 3600 Hz; pulse width, 4.0 μs; relaxation delay, 1 s; and acquiring time, 2.67 μs. For quantitative analysis, the peak area was used and the start and end points of the integration of each peak were selected manually.

### 2.3 Sample preparation

For extraction, 0.1 g of EGB761 sample was dissolved in 30 mL of 5% HCl, heated to reflux for 30 min, and then partitioned three times with cyclohexane (30 mL). The remaining water layer was partitioned by methylethyl ketone (30 mL × 3 times). The methylethyl ketone fractions were evaporated and dissolved in 0.6 mL of a mixture of methanol‐d4 and benzene‐d6 (65:35). All experiments were performed in triplicate.

### 2.4 Materials and animals

All procedures performed were approved by the Subcommittee on Research‐Animal Care of the National Cheng Kung University Medical Center, and in agreement with the guidelines of the Taiwan National Institutes of Health. Animal experiments followed the approved protocol (IACUC approval no. 112163). EGb761 was provided by Dr. Willmar Schwabe GmbH & Co. KG (Karlsruhe, Germany). All chemicals, unless otherwise stated, were purchased from Sigma‐Aldrich Co. (St. Louis, MO, USA).

### 2.5 In vitro experiments

#### 2.5.1 2,2′‐diphenyl‐1‐picrylhydrazyl radical scavenging assay

The diphenyl‐1‐picrylhydrazyl (DPPH) radical scavenging assay was conducted following a previously published procedure (Kraus et al., 2005). DPPH radicals (D9132, Sigma‐Aldrich Co., St. Louis, MO, USA) were dissolved in dimethyl sulfoxide (DMSO) to achieve a final concentration of 100 μM. The reactions were initiated by adding 100 μL of freshly prepared DPPH radical solution to 100 μL of compounds at various concentrations, dissolved in 0.1% DMSO. Incubation took place at room temperature (25°C) for 30 min in the absence of light. Absorbance measurements were performed at 517 nm using a plate reader (Stat Fax 2100).

#### 2.5.2 2,2′‐azino‐bis (3‐ethylbenzothiazoline‐6‐sulfonic acid) diammonium salt (ABTS) radical scavenging assay

The ABTS radical scavenging activity was determined as previously described (Miller & Rice‐Evans, 1997). In brief, ABTS radical cations (A1888, Sigma‐Aldrich Co., St. Louis, MO, USA) were prepared by reacting 7 mM ABTS with 2.5 mM potassium persulfate in water, under dark conditions, overnight. This solution was then diluted with phosphate‐buffered saline (PBS) at pH 7.4 to achieve a final concentration of 20 mM potassium phosphate and 50 mM NaCl. Reactions were initiated by adding 100 μL of the ABTS radical cation solution to 100 μL of the compound in 0.1% DMSO. Similar to the DPPH assay, the reactions were incubated at room temperature (25°C) for 30 min in the absence of light, and absorbance was measured at 734 nm using a plate reader (Stat Fax 2100).

#### 2.5.3 Cell cultures for RAW 264.7 and immune response induction

The immortalized murine macrophage cell line RAW 264.7 (ATCC TIB‐71TM) was maintained at a density of 1 × 10^6^ cells/mL in DMEM supplemented with 10% heat‐inactivated fetal bovine serum (A5256701, Gibco, Thermo Fisher Scientific Inc., USA) and incubated at 37°C in a humidified atmosphere containing 5% CO_2_ and 95% air (Lee et al., 2009). On the following day, the medium was replaced with fresh DMEM, and cells were stimulated with Lipopolysaccharide (LPS, 100 ng/mL) while co‐treated with either a vehicle (PBS) or EGb761 (25–1000 μg/mL). Culture supernatants were collected for the measurement of tumor necrosis factor‐alpha (TNF‐α) and interleukin‐6 (IL‐6) levels after incubation for 6 h and for the assessment of nitrite/nitrate levels after incubation for 16 h. All experiments were conducted in duplicate with assays performed in triplicate (Lee et al., 2009).

#### 2.5.4 Cell toxicity testing (MTT assay)

MTT (3‐(4,5‐dimethyl‐2 thiazoyl)‐2,5‐diphenyl‐tetrazolium bromide) (475, 989, Sigma‐Aldrich Co., St. Louis, MO, USA), was dissolved in PBS to prepare a 5 mg/mL stock solution. Cells to be tested were cultured in a 96‐well plate, and after 24 h of pre‐incubation with EG761 (25–1000 μg/mL), MTT was added. The MTT was mixed with the samples at a 1:5 ratio and incubated in the dark for 3 h. Subsequently, 100 μL of DMSO was used to dissolve the formazan crystals. Absorbance was measured at 532 nm using an enzyme immunoassay analyzer.

#### 2.5.5 IL‐6, TNF‐α, and nitrate/nitrite assay

Levels of IL‐6 and TNF‐α in culture supernatants were determined using an enzyme‐linked immunosorbent assay (ELISA) method. A DuoSet ELISA development system for mouse IL‐6 (DY406, R&D Systems, Minneapolis, MN, USA) and TNF‐α (DY410, R&D Systems, Minneapolis, MN, USA) was employed for the ELISA. Nitric oxide (NO) production was assessed by measuring nitrate/nitrite accumulation in the culture medium using a nitrate/nitrite fluorometric assay kit (780,051, Cayman Inc., Ann Arbor, MI, USA). TNF‐α and IL‐6 levels were determined by measuring the optical density at 450 nm with a plate reader (Stat Fax 2100), and nitrate/nitrite levels were assessed with a Fluoroskan Ascent FL microplate reader (Thermo Electron Co., Milford, MA, USA) at an excitation wavelength of 360–365 nm and an emission wavelength of 430 nm.

### 2.6 In vivo experiments

#### 2.6.1 Limb ischemia animal model

Male Sprague–Dawley rats weighing 250–300 g were obtained from the Animal Center at National Cheng Kung University Hospital. Rats were allowed free access to food and water before and after surgery. Prior to surgery, rats were anesthetized with intraperitoneal injection of chloral hydrate. Anesthesia was maintained using a temperature‐controlled heating pad, and rectal temperature was monitored using a rectal probe (Harvard Apparatus, South Natick, MA) to maintain the body temperature at 37 ± 0.5°C. Body temperature control was achieved by placing a heating pad under the abdominal region of the animals and inserting a temperature probe into the rectum to continuously monitor body temperature. Thirty minutes before ischemia induction, intravenous injections were administered via the femoral vein. The injections included vehicle (normal saline), and EGb761 at doses of 25, 50, 100, and 200 mg/kg. A tourniquet was applied to the hind limb of the S.D. rat to induce 2 h of ischemia. An ICP transducer (Codman MicroSensor) was inserted under the tourniquet to monitor the applied pressure, maintained at approximately 350–380 mmHg. After 2 h of ischemia, the tourniquet was released, and 24 h of reperfusion followed. A PeriScan PIM 3 System (Perimed AB, Sweden) was employed to record local blood flow in the hind limb muscles of the S.D. rats, covering five‐time points: pre‐ischemia, post‐ischemia, pre‐reperfusion, post‐reperfusion, and post‐reperfusion at 24 h.

### 2.7 Blood flow measurement

Blood flow measurements were conducted using the non‐invasive PeriScan PIM 3 System (Perimed AB), which employs laser Doppler imaging to visualize blood flow dynamics in superficial tissues. The extent of the success of transient local hind limb ischemia and subsequent reperfusion was primarily based on the percentage of hind limb muscle blood flow relative to baseline prior to ischemia induction. The blood flow was reduced to approximately 10% of baseline during the hind limb ischemia phase, and during the reperfusion phase, it needed to recover to more than 50% of baseline to be considered successful. This assessment was consistently performed across all experimental subjects to ensure uniformity of the hind limb ischemic insult. Blood flow measurements were taken at five‐time points: pre‐ischemia, post‐ischemia, pre‐reperfusion, post‐reperfusion, and post‐reperfusion at 24 h. During measurements, care was taken to ensure rats were fully anesthetized, as any movement could interfere with the accuracy of the data obtained by the equipment. Following completion of the entire surgical procedure, rats were returned to their cages, provided warmth, and given ample food and water until the subsequent experimental stages.

### 2.8 Measurement of GSH levels

To assess glutathione (GSH) levels, the muscle tissue was initially weighed and then homogenized in a solution containing 0.1 M Na_2_HPO_4_ and 5 mM EDTA, achieving a 1:20 ratio for homogenization. The resulting homogenate was centrifuged at 20,000*g* for 30 min at a temperature of 4°C. Both the sample and a standard were taken at 100 μL combined with 900 μL of Na_2_HPO_4_‐EDTA buffer. o‐phthalaldehyde (OPA, 79760, Sigma Chemical Co.; St. Louis, MO, USA) at a concentration of 5 mg/mL was added 50 μL to each of these mixtures were then incubated in the dark at room temperature for a duration of 15 min. Subsequently, 200 μL of each incubated mixture was measured using an enzyme immunoassay fluorescence analyzer. The absorbance values were recorded at an excitation wavelength of 350 nm and an emission wavelength of 420 nm.

### 2.9 Myeloperoxidase activity

Myeloperoxidase (MPO) activity was used to quantify the neutrophil infiltration by the method described previously (Pruitt et al., 1990). Blood was extracted from rats with heparin‐rinsed equipment. ACK lysis buffer was added (blood: ACK lysis buffer = 1:8 ratio), and the mixture was incubated on ice for 6 min. Subsequently, it was centrifuged at 800*g* for 5 min at 4°C. Repeated 1–2 times to eliminate red blood cells. The cell pellet was washed with PBS, and protease and phosphatase inhibitors were added. Next, 3 mL of 0.5% CTAB (219, 374, Merck‐KgaA; Darmstadt, Germany) was added and kept on ice for 5 min. The mixture was then centrifuged at 12,000*g* for 15 min at 4°C, and the supernatant was collected as the sample. For the MPO activity assay, 30 μL of the sample was mixed with 170 μL of 0.02% CTAB and different concentrations of the test compounds, followed by an incubation period of 3–5 min. Subsequently, 2 μL of Guaiacol/DMSO and 2 μL of 1% H_2_O_2_ were added, and the mixture was shaken. It is important to note that the reaction time must be consistent. The absorbance was measured at 450 nm.

### 2.10 Lipid peroxidation assay

Skeletal muscle tissue sample (50 μL) or standard solution (50 μL) was reacted with a mixture consisting of 300 μL of 1% phosphoric acid and 100 μL of 0.6% TBA. The reaction mixture was incubated at 95°C for 60 min, and after incubation, the reaction was halted by distilled water, followed by the addition of 400 μL of N‐butanol. The resulting mixture was vigorously shaken and centrifuged at 2000 rpm for 10 min. Absorbance was measured at 532 nm. Tissue malondialdehyde (MDA) levels, indicative of lipid peroxidation, were calculated from the standard curve and expressed as nmol/mg of protein.

### 2.11 Nitrate/nitrite assay

Before and 24 h after ischemia–reperfusion injury (IRI), blood samples were collected from the right jugular vein for the determination of nitrate/nitrite levels. NO production was assessed using a nitrate/nitrite fluorometric assay kit (780, 051, Cayman Inc., Ann Arbor, MI). Following the vendor's instructions, nitrate/nitrite assays were conducted using a Fluoroskan Ascent FL microplate reader (Thermo Electron Co., Milford, MA) at an excitation wavelength of 360–365 nm and an emission wavelength of 430 nm.

### 2.12 Histological evaluation and cross‐sectional area analysis

Muscle samples were obtained 24 h after ischemia–reperfusion injury and placed in 10% formaldehyde. Following paraffin embedding, 5 μm tissue sections were stained with hematoxylin and eosin (H&E) for histological evaluation. Subsequently, these sections were examined under a Zeiss Axioskop 2 Mot microscope equipped with a digital CoolSnap‐Pro cf camera (Media Cybernetics, Inc., Carlsbad, CA) and a semi‐automated image analysis system (MCID Elite) for measurements. To quantify intact muscle fiber size, we utilized the MCID Elite system to analyze the muscle cross‐sections. Briefly, captured images were imported into the software. Individual muscle fibers were outlined, and their cross‐sectional area (CSA) was calculated. To minimize bias, a minimum of six non‐overlapping fields per muscle section were analyzed. The average CSA of muscle fibers was then determined for each sample.

### 2.13 Immunohistochemistry

The muscle sections were collected on poly‐L‐lysine coated slides for further processing. For immunohistochemistry, tissue sections were deparaffinized and rehydrated the tissue by xylene and ethanol. The tissue section was in a preheated buffer solution (citrate buffer, pH 6.0) heated at 95–100°C for 10–20 min, and immersed in blocked buffer (10% horse serum, 1% triton X‐100, in PBS) for 1 h. After blocking, sections were incubated with MPO antibody (1:100; RB‐373, Thermo Fisher Scientific, Fremont, USA), and Macrophage (1:100; MCA874GA, AbD serotec a division of MorphoSys, MorphoSys UK) at 4°C overnight. Appropriate secondary antibody conjugated with biotin (1:100) was then added, followed by peroxidase‐conjugated streptavidin (1:100) (Jackson ImmunoResearch, Inc., West Grove, PA, USA). Sections were visualized under a microscope (Olympus IX71; Olympus Optical Co., Ltd., Tokyo, Japan). The photos were analyzed by Image Pro Plus 5.1 software (Media Cybernetics, Silver Spring, MD, USA).

### 2.14 Statistical analysis

Data were analyzed using one‐way ANOVA followed by Fisher's protected least significant difference (LSD) post‐hoc or Student's *t*‐test to detect significant differences between the means with *p* < 0.05. Data were presented as the mean ± standard deviation (SD). Analysis of data was performed using SPSS Statistics for Windows, version 17.0 (SPSS Inc., Chicago, IL, USA).

## 3 RESULTS

**FIGURE 1:**
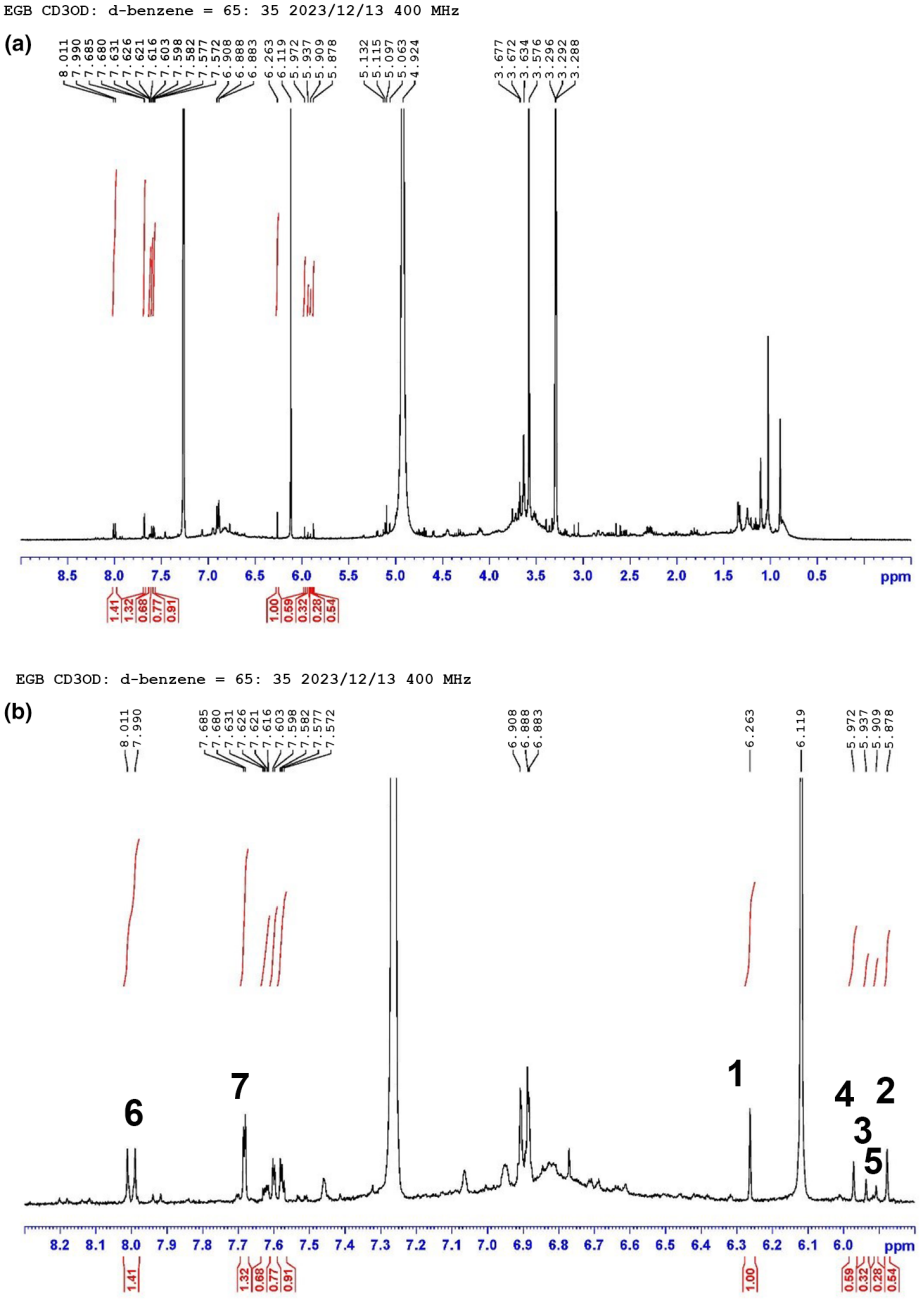
1H NMR spectra of EGB sample. (a) Full 1H NMR spectrum of EGB sample; (b) Expanded region of 5.8–8.3 ppm for the 1H NMR spectrum of EGB sample. Compounds **1**–**7** were quantified according to the peaks numbered.

**FIGURE 2:**
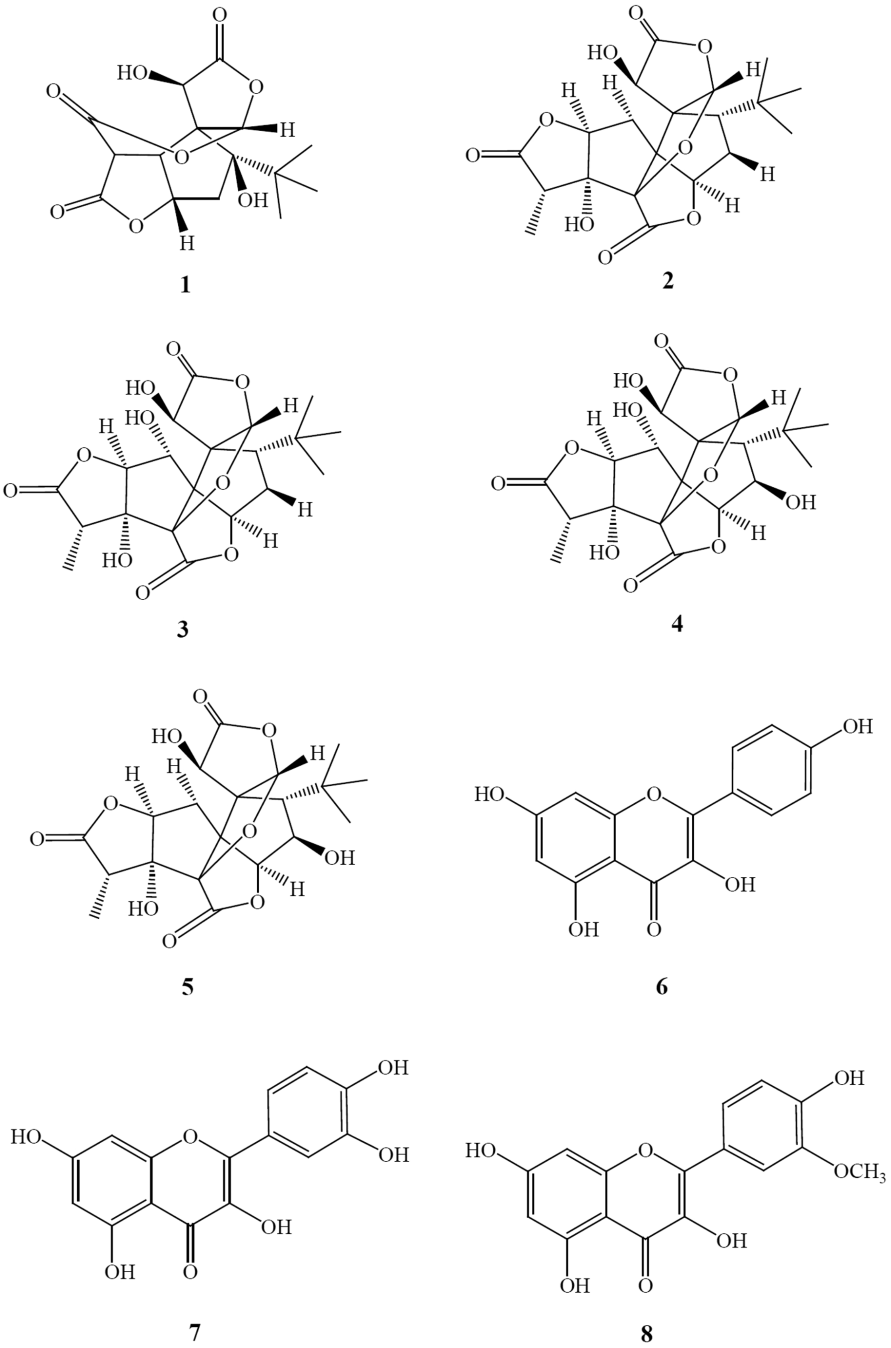
Structures of compounds **1**–**8**. Seven compounds could be identified, including bilobalide A (**1**), ginkgolides A–C (**2**–**4**), −J (**5**), kaempferol (**6**), and quercetin (**7**) but not Isorhamnetin (**8**).

The EGB sample was yielded 1H NMR spectra as shown in Figure 1. In comparison of the present chemical shifts with those reported by us, the following seven compounds could be identified, included bilobalide A (**1**), ginkgolides A‐C (**2**–**4**), −J (**5**), kaempferol (**6**), and quercetin (**7**) (Figure 2). Isorhamnetin (**8**) (Figure 2) could not be observed in the EGB sample maybe due to its presence being lower than the limit of detection (LOD). The quantification results of compounds **1**–**7** were provided in Table 1.

**TABLE 1:** Quantification results of compounds **1**–**7** (mg/50 mg extract) in EGB sample.

Compound	**1**	**2**	**3**	**4**	**5**	**6**	**7**
	1.86	1.57	0.84	1.36	1.25	2.54	2.53

### 3.1 In vitro experiments

#### 3.1.1 Radical scavenging assays and cell viability assessment

**FIGURE 3:**
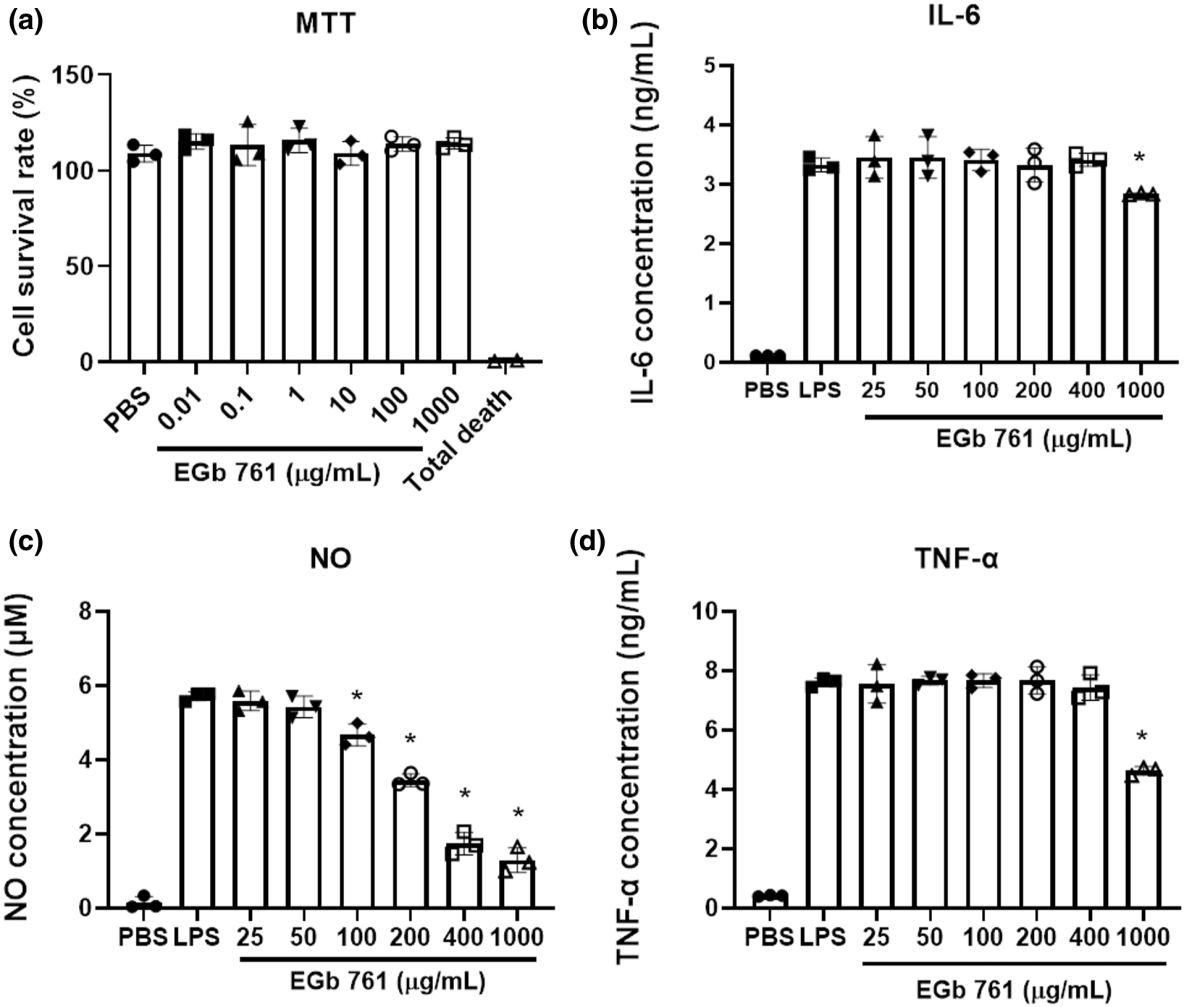
The cytotoxicity and anti‐inflammatory capabilities of EGb761 in the immortalized murine macrophage RAW 264.7. (a) In the MTT assay, EGb761 (0.01–1000 μg/mL) exhibited minimal cytotoxicity, indicating a safe dosage range for this compound. Inhibitory effects of EGb961 on interleukin‐6 (IL‐6), tumor necrosis factor‐alpha (TNF‐α) and nitric oxide (NO) production in LPS‐treated RAW264.7 cells. The cells were stimulated with lipopolysaccharide (LPS; 100 ng/mL) and co‐treated with EGb761 (25–1000 μg/mL) (b–d) the levels of IL‐6, TNF‐α and NO in the supernatants were measured. The mean ± SD expresses data, *n* = 3, *p > 0.05, compared with the LPS group.

EGb761 was effectively active in an ABTS radical cation scavenging assay, and EGb761 was effective with an IC50 value of 0.016 mg/mL (Table 2). In addition, EGb761 was also active in DPPH radical scavenging assay with an IC50 value of 1.609 mg/mL (Table 2). To assess the cytotoxicity of EGb761, we conducted a cell viability assessment (MTT Assay) of various concentrations of EGb761 on raw264.7. The cell viability of the PBS group as the baseline and 0.8% Triton X‐100 as a representation of total cell death. EGb761 was administered at different concentrations (0.01–1000 μg/mL) to evaluate its impact on cell viability. The cell survival rate in each EGb761 group (0.01–1000 μg/mL) revealed minimal differences compared to the PBS group and had no statistically significant variance (Figure 3a, *p* > 0.05). These results indicate that treatment with varying concentrations of EGb761 does not induce cytotoxicity in these cells.

**TABLE 2:** Effective concentration (IC50) in scavenging free radicals.

	IC_50_ (μM)
ABTS	
α‐Tocopherol	14.4 ± 1.8
Ascorbic acid	23.3 ± 0.6
EGb 761	16.0 ± 1.0
DPPH	
α‐Tocopherol	168.2 ± 0.8
Ascorbic acid	73.3 ± 1.2
EGb 761	1609.0 ± 50.0

*Note*: Data are represented as the mean ± standard deviation (SD). The IC50 value was defined as the concentration of compound required to reduce the absorbance to one‐half of the initial value in 30 min at 37°C, *n* = 4.

#### 3.1.2 Effects of EGb761 on proinflammatory cytokines and NO production in LPS‐stimulated RAW 264.7 cells

Based on the result of MTT assay data, it was determined that a concentration of 1000 μg/mL EGb761 does not induce cellular toxicity. Therefore, this concentration was employed in the measurement of the immunomodulatory cytokines IL‐6 and TNF‐α in immune cells. Following a 6‐h stimulation of RAW 264.7 cells with LPS, the levels of IL‐6 and TNF‐α were assessed, employing the concentrations observed in the PBS group as the reference baseline. EGb761 significantly reduced proinflammatory cytokines IL‐6 and TNF‐α production in LPS‐stimulated RAW 264.7 cells, respectively (IL‐6, *p* = 0.002; TNF‐α, *p* = 0.0016; Figure 3b,c). In addition, NO production was assessed by subjecting RAW 264.7 cells to LPS stimulation for 16 h, followed by the evaluation of NO levels. The concentrations in the PBS group were utilized as the baseline for comparison. EGb761 significantly reduced NO production in LPS‐stimulated RAW 264.7 cells (EGb 761,100, 200, 400, 1000 μg/mL, *p* = 0.0048, 0.0000, 0.0000, 0.0000; Figure 3d).

### 3.2 In vivo experiments

#### 3.2.1 Local limb blood flow monitoring during ischemia

**FIGURE 4:**
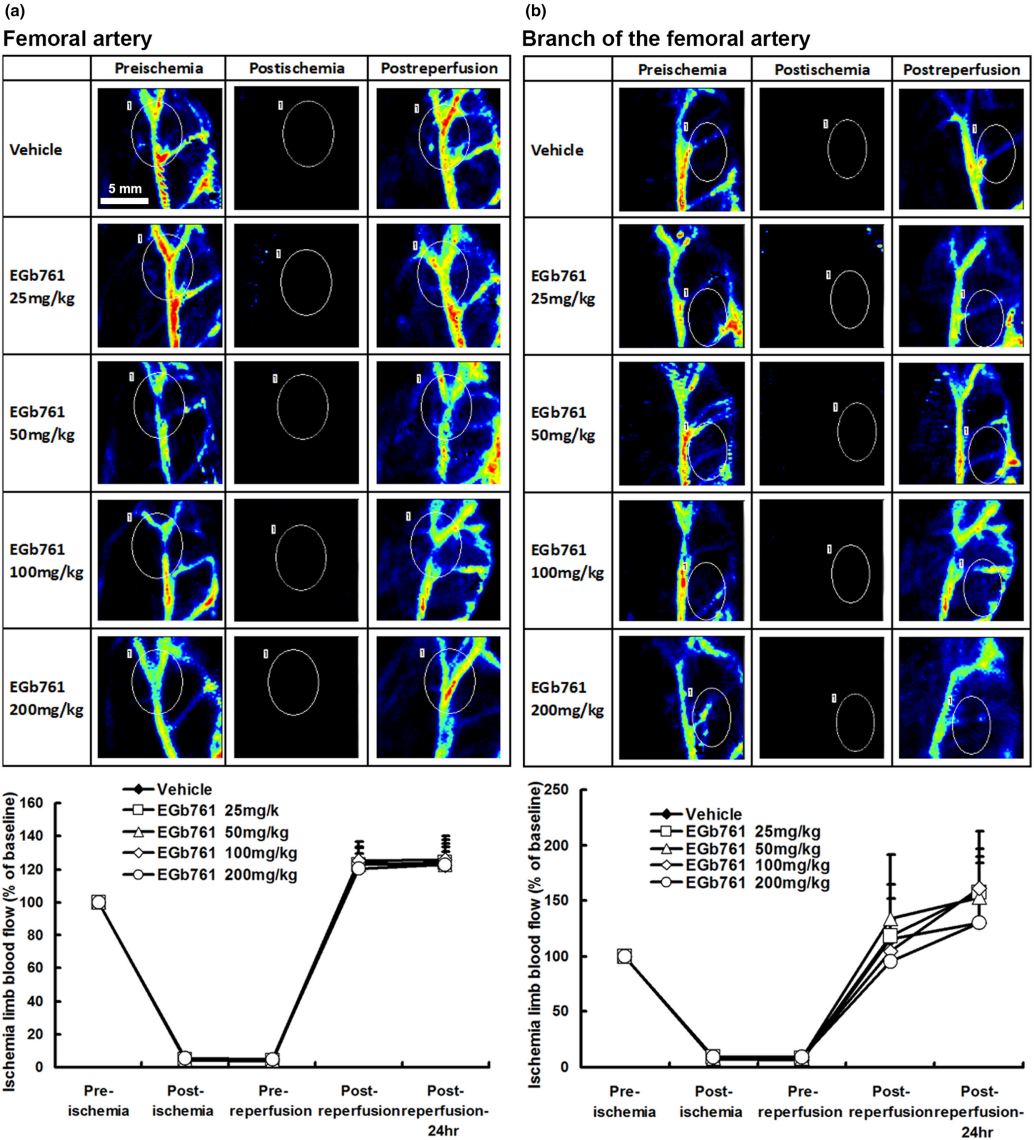
Local muscular femoral artery and branch blood flow monitoring. The femoral artery and branch blood flow were assessed by scanning laser Doppler imaging prior to and during ischemia and post‐reperfusion. Pre‐treatment of Vehicle (normal saline) or EGb761 (25–200 mg/kg) did not influence the blood flow in (a) femoral artery or (b) branch in each group, *n* = 10.

Local limb blood flow parameters in hindlimb ischemia were measured relative to baseline measurements. The parameters obtained at each time point, including pre‐ischemia (baseline), post‐ischemia, pre‐reperfusion, post‐reperfusion, and post‐reperfusion‐24 h, were expressed as relative percentages. The blood flow of each group was also displayed in a laser Doppler image (Figure 4).

There were no significant statistical differences between the five groups in terms of the main femoral artery (Figure 4a) and collateral artery (Figure 4b) blood flow at any time point during the experiment (*p* > 0.05). This suggests that the protective effect of EGb761 in limb ischemia–reperfusion is not mediated by collateral blood flow. Additionally, in all five groups, local blood flow decreased to less than 10% of the initial value after muscle ischemia, and it recovered to over 90% after reperfusion. This confirms that the ischemia–reperfusion monitoring process was stable and successful for all groups, regardless of whether they were pretreated with normal saline or EGb761 at different concentrations.

#### 3.2.2 The protective effects of EGb761 against ischemic injury to muscle fibers

**FIGURE 5:**
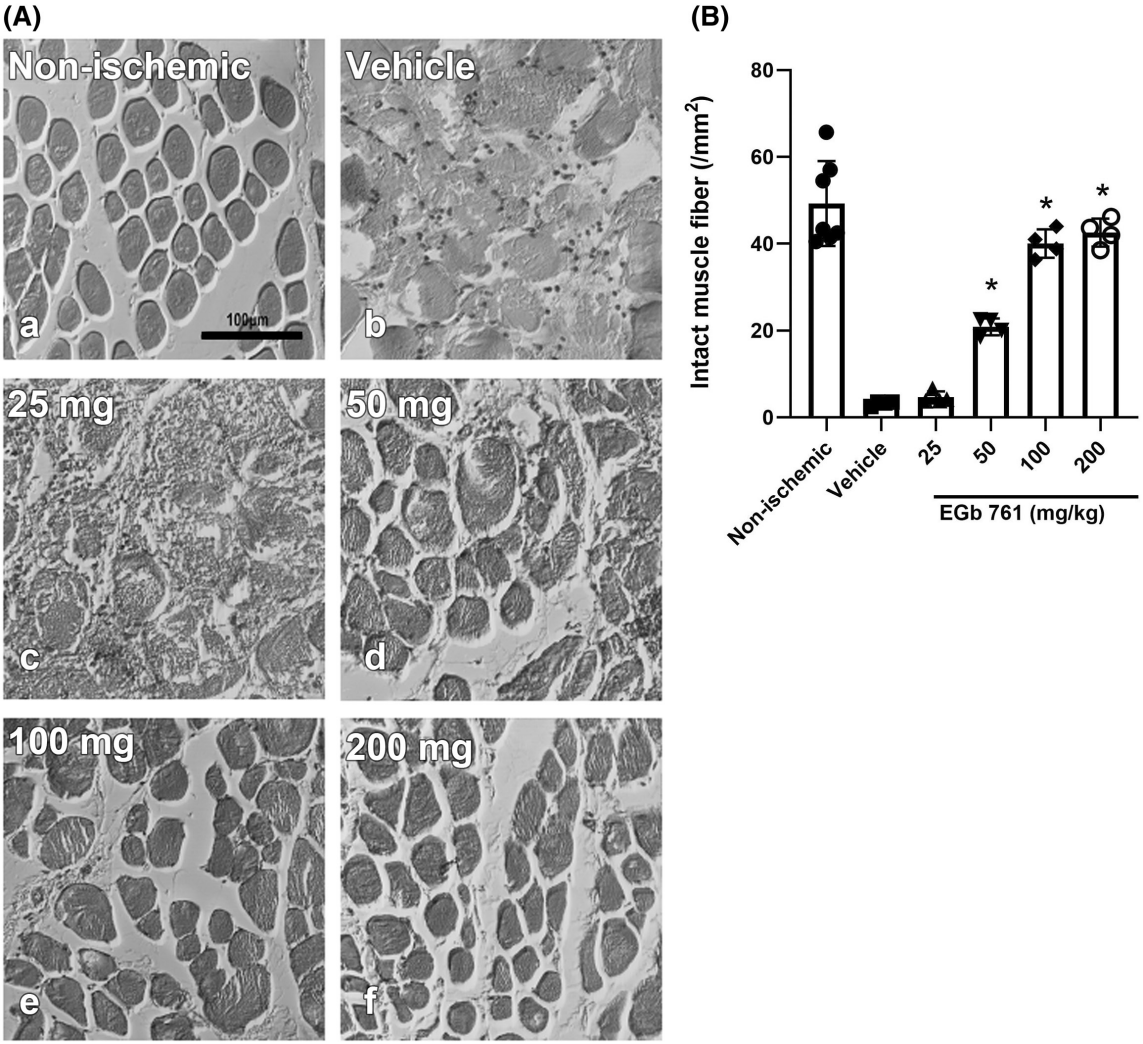
EGB761 reduces muscle fiber damage after ischemia–reperfusion injury. The rats suffered 2 h of limb ischemia and treatment with vehicle and EGb761 (0–200 mg/kg) at reperfusion onset. After 24 h limb skeletal muscle was sampled for Hematoxylin–eosin staining. (A) Skeletal muscle fibers were observed at 200× to observe the differences that showed injured muscle fibers with atypical morphology. (B) The region of intact muscle fiber remained more in EGb761 treatment groups. The mean ± SD expresses data, *n* = 4–7, *p > 0.05 compare with vehicle.

Rats were pretreated with normal saline or various doses of EGb761 for 24 h. After ischemia–reperfusion for 24 h, skeletal muscle from the hindlimbs was harvested for hematoxylin and eosin staining. The intact skeletal muscle fiber percentage was assessed by three independent observers. Skeletal muscle fibers in the non‐ischemic group were well‐organized and intact (Figure 5Aa). In the vehicle group, skeletal muscle fibers were severely damaged and disorganized (Figure 5Ab). EGb761 25 mg/kg did not significantly improve the morphology of skeletal muscle fibers compared with the vehicle group (Figure 5Ac). The percentage of intact skeletal muscle fibers was significantly improved in EGb761 50, 100, and 200 mg/kg compared with the vehicle group (Figure 5Ad–f). The percentage of intact skeletal muscle fibers was significantly higher in the EGb761 50, 100, and 200 mg/kg groups than in the vehicle group (Figure 5b, EGb 761 50, 100, 200 mg/kg, *p* = 0.0000, 0.0000, 0.0000).

#### 3.2.3 Effects of EGb761 on GSH and NO production in skeletal muscle fiber following ischemia–reperfusion injury

**FIGURE 6:**
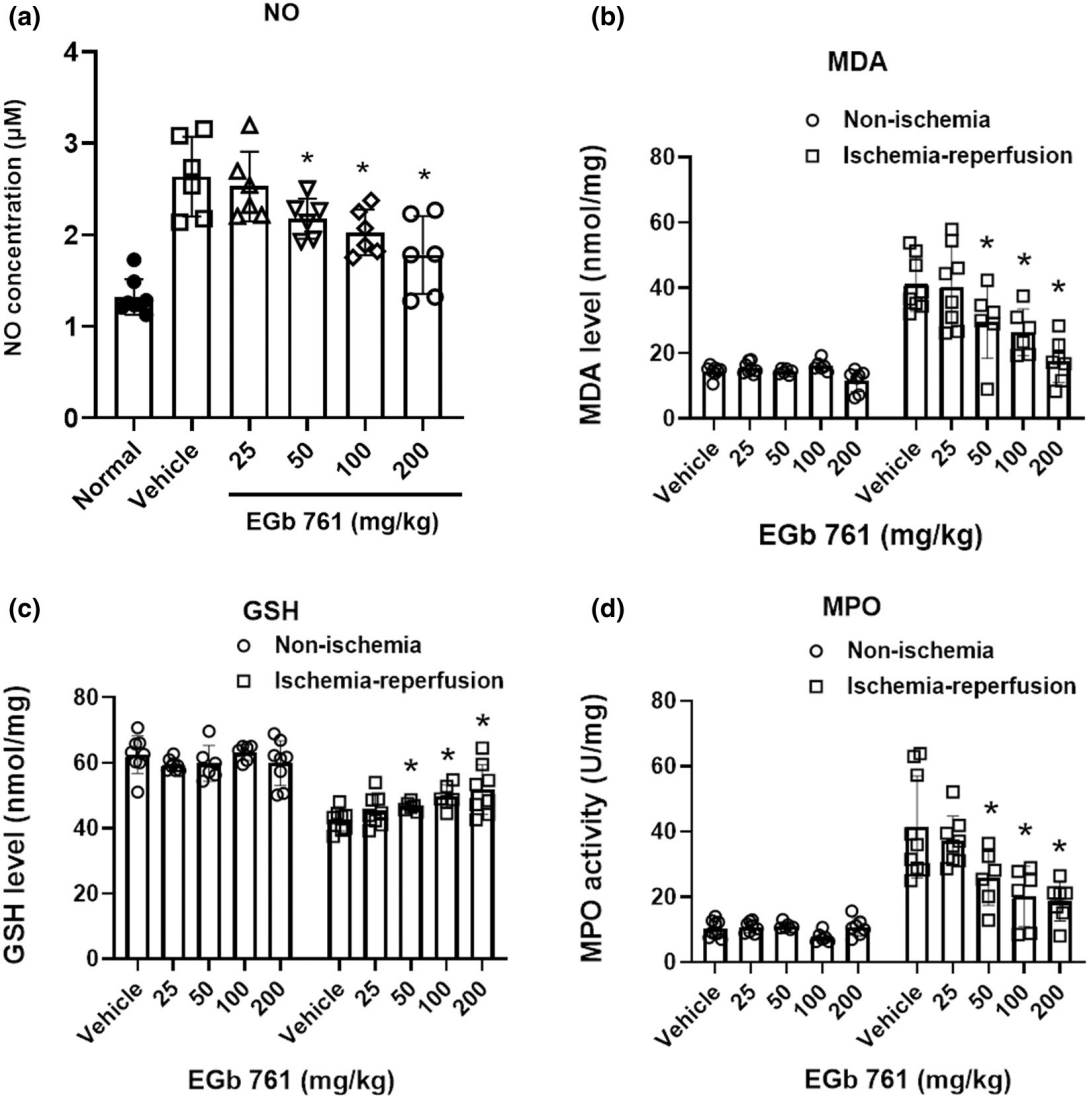
The anti‐oxidative and anti‐inflammatory effects of EGb761 in the skeletal muscle following limb ischemia–reperfusion injury. The rats suffered 2 h of limb ischemia and treatment with vehicle and EGb761 (25–200 mg/kg) at reperfusion onset. After 24 h serum sampling to assess (a) nitric oxide (NO) and limb skeletal muscle was sampled to assess the level of (b) lipid peroxidation, (c) glutathione (GSH), and (d) neutrophil infiltrations. The mean ± SD expresses data, *n* = 6–8, *p > 0.05, compared with vehicle.

The animal hindlimb was subjected to ischemia–reperfusion injury. After 24 h, blood serum was collected to measure NO production and muscle samples were collected to measure GSH, MDA, and MPO levels. EGb761 treatment at 50–200 mg/kg significantly reduced NO production in serum following ischemia–reperfusion (EGb 761 50, 100, 200 mg/kg, *p* = 0.043, 0.013, 0.006, Figure 6a). In the non‐ischemia limb, there was no significant difference in MPO, MDA, or GSH levels between the groups. In the ischemia–reperfusion injured limb, EGb761 treatment at 50–200 mg/kg significantly increased GSH levels (an important antioxidant) (EGb 761 50, 100, 200 mg/kg *p* = 0.146, 0.002, 0.007; Figure 6b) and reduced MDA production (inhibiting lipid peroxidation) respectively (EGb 761 50, 100, 200 mg/kg *p* = 0.033, 0.005, 0.000; Figure 6c). This suggests that EGb761 can help to protect muscle fibers from oxidative damage. Furthermore, EGb761 at 50–200 mg/kg significantly inhibited MPO activity (EGb 761 50, 100, 200 mg/kg *p* = 0.046, 0.011, 0.003; Figure 6d), indicating that it can reduce inflammation and protect muscle fibers.

#### 3.2.4 EGb761 reduces immune cell activation in skeletal muscle ischemia–reperfusion injury

**FIGURE 7:**
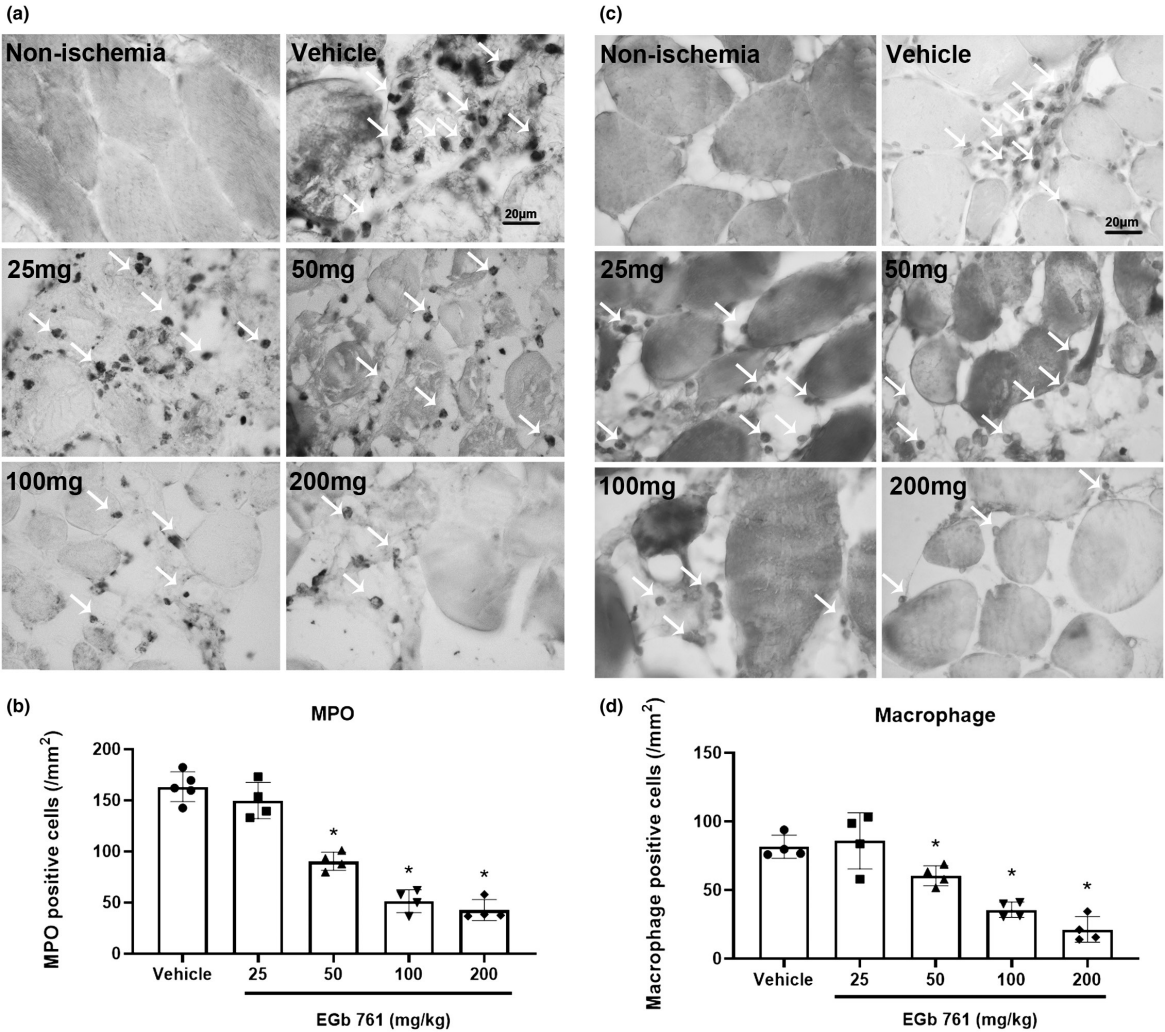
The anti‐inflammatory effect of EGb761 in skeletal muscle by reducing activated MPO‐positive cells and macrophage. The rats suffered 2 h of limb ischemia and treatment with vehicle and EGb761 (25–200 mg/kg) at reperfusion onset. Immunohistochemical staining of (a) MPO and (c) macrophages in skeletal muscle at 24 h after ischemia–reperfusion injury. (b) MPO staining showed decreased expression in the EGb761 groups compared to the vehicle group. (d) Macrophage staining showed a decreased number of cells in the EGb761 groups compared to the vehicle group. Mean ± SD, *n* = 4, *p > 0.05, compared with vehicle.

Immunohistochemistry (IHC) staining was performed on skeletal muscle samples from the non‐ischemic and ischemic‐reperfusion limbs. The infiltration of immune cells, including neutrophils (Figure 7a) and macrophages (Figure 7c), was evaluated in the skeletal muscle tissue of the vehicle and EGb761 groups. In our results, EGb761 treatment at 50–200 mg/kg significantly decreased the number of MPO‐positive cells in the muscle tissue (EGb 761 50, 100, 200 mg/kg *p* = 0.000, 0.000, 0.000; Figure 7b). In addition, the number of macrophage cells was also significantly reduced in the EGb761 50–200 mg/kg groups (EGb 761 50, 100, 200 mg/kg *p* = 0.009, 0.000, 0.000; Figure 7d).

## 4 DISCUSSION

EGb761, generally considered to have milder and fewer side effects due to its natural properties, has been found in previous studies to have various beneficial effects on the organism. These include clearing free radicals, reducing oxidative stress, minimizing neural damage, decreasing platelet aggregation, and exhibiting anti‐inflammatory and anti‐tumor activities (Chan et al., 2007). In past experiments, it has been discovered that muscle damage after posterior lumbar spine surgery is long‐lasting (Kawaguchi et al., 1994b). The operation time and retraction pressure applied during surgery are considered a contributing factor to muscle damage (Kawaguchi et al., 1994a). Previous literature has mentioned that potential causes of muscle atrophy following spinal surgery include iatrogenic denervation and direct muscle damage resulting from dissection or retraction during the procedure (Cho et al., 2020). These factors may lead to subsequent postoperative pain, adjacent segment disease, and kyphosis. Minimizing ischemia–reperfusion injury caused by the use of retractors may reduce muscle damage and complications following lumbar surgery (Hori et al., 2013).

In our study, we investigated the effects of EGb761 on inflammation and tissue damage in both in vitro and in vivo models, focusing on ischemia–reperfusion injury. In vitro experiments with the RAW 264.7 cell line demonstrated that EGb761 at a concentration of 1000 μg/mL is non‐cytotoxic and effectively reduces IL‐6, TNF‐α concentrations, and NO production in a dose‐dependent manner compared to LPS‐induced cells. This reduction in pro‐inflammatory cytokines suggests that EGb761 has a significant anti‐inflammatory effect.

In vivo, pre‐administration of EGb761 showed no significant changes in femoral artery blood flow, indicating that its protective effect against ischemia–reperfusion injury does not stem from altered collateral blood flow. However, substantial improvements in skeletal muscle integrity and reduced inflammation were observed, suggesting that EGb761 might protect against ischemia–reperfusion injury through different mechanisms.

When administered before the ischemia–reperfusion event, EGb761 exhibited a linear, dose‐dependent protective effect on tissue histopathology, immune tissue staining (MPO and macrophages), reduced oxidative stress, inflammatory response, and lipid peroxidation. These results suggest that EGb761 has both anti‐inflammatory and anti‐oxidative effects.

It is crucial to delineate how these effects manifest differently in macrophages versus muscles. The anti‐inflammatory effect is likely due to the reduction of inflammatory cytokines in macrophages, justifying their inclusion in the experimental methods. On the other hand, the anti‐oxidative effect could directly protect muscles from oxidative stress, influencing muscle‐specific force independently of muscle mass. These distinctions can be significant for understanding how EGb761 contributes to reduced tissue damage and inflammation.

Past research on EGb761 has predominantly focused on its antioxidant and anti‐inflammatory effects on the brain and heart (Barbalho et al., 2022; Defeudis & Drieu, 2000; Hu et al., 2003; Shen et al., 1998). This study represents the first attempt to apply EGb761 in reducing ischemia–reperfusion injury in skeletal muscle. Previous literature has utilized free radical scavenger drugs to mitigate ischemia–reperfusion injury (Hori et al., 2013); however, these medications tend to be expensive and difficult to obtain. By employing EGb761, which is more accessible and cost‐effective, we leveraged its anti‐inflammatory and antioxidant properties to decrease ischemia–reperfusion injury in skeletal muscle.

## AUTHOR CONTRIBUTIONS

*Conceptualization*: E‐Jian Lee, Shih‐Huang Tai. *Methodology*: Shih‐Huang Tai and Liang‐Yi Chen. *Validation*: Shih‐Huang Tai and Liang‐Yi Chen. *Formal analysis*: Yu‐Chang Hung, Hao‐Hsiang Hsu. *Investigation*: Sheng‐Yang Huang, Zi‐Cheng Kuo. *Resources*: Tian‐Shung Wu. *Data curation*: Ai‐Hua Lee. *Writing*—*original draft preparation*: Liang‐Yi Chen and Sheng‐Yang Huang. *Writing*—*review and editing*: E‐Jian Lee. *Visualization*: Hao‐Hsiang Hsu and Zi‐Cheng Kuo. *Supervision*: E‐Jian Lee. Project administration: Shih‐Huang Tai. All authors have read and agreed to the published version of the manuscript.

## FUNDING INFORMATION

The present study was funded by a grant from the National Cheng Kung University Hospital: NCKUH‐9902062.

## CONFLICT OF INTEREST STATEMENT

The authors declare no conflict of interest.

## ETHICS STATEMENT

All procedures performed were approved by the Subcommitteeon Research‐Animal Care of the National Cheng Kung University Medical Center, and in agreement with the guidelines of the Taiwan National Institutes of Health. Animal experiments followed the approved protocol (IACUC approval No. 112163).

## Data Availability

The data that support the findings of this study are available from the corresponding author upon reasonable request.
